# Supplementary material for: Effects of a High-Concentrate Diet on the Blood Parameters and Liver Transcriptome of Goats
Source: Animals (Basel). 2023 May 6;13(9):1559. doi: 10.3390/ani13091559 (PMC10177143; doi:10.3390/ani13091559)
Supplement: Supplementary file 1 [file animals-13-01559-s001.zip › animals-2263165-supplementary.pdf]

animals

MDPI

Supplementary materials

1. Figures

A

B

C

D

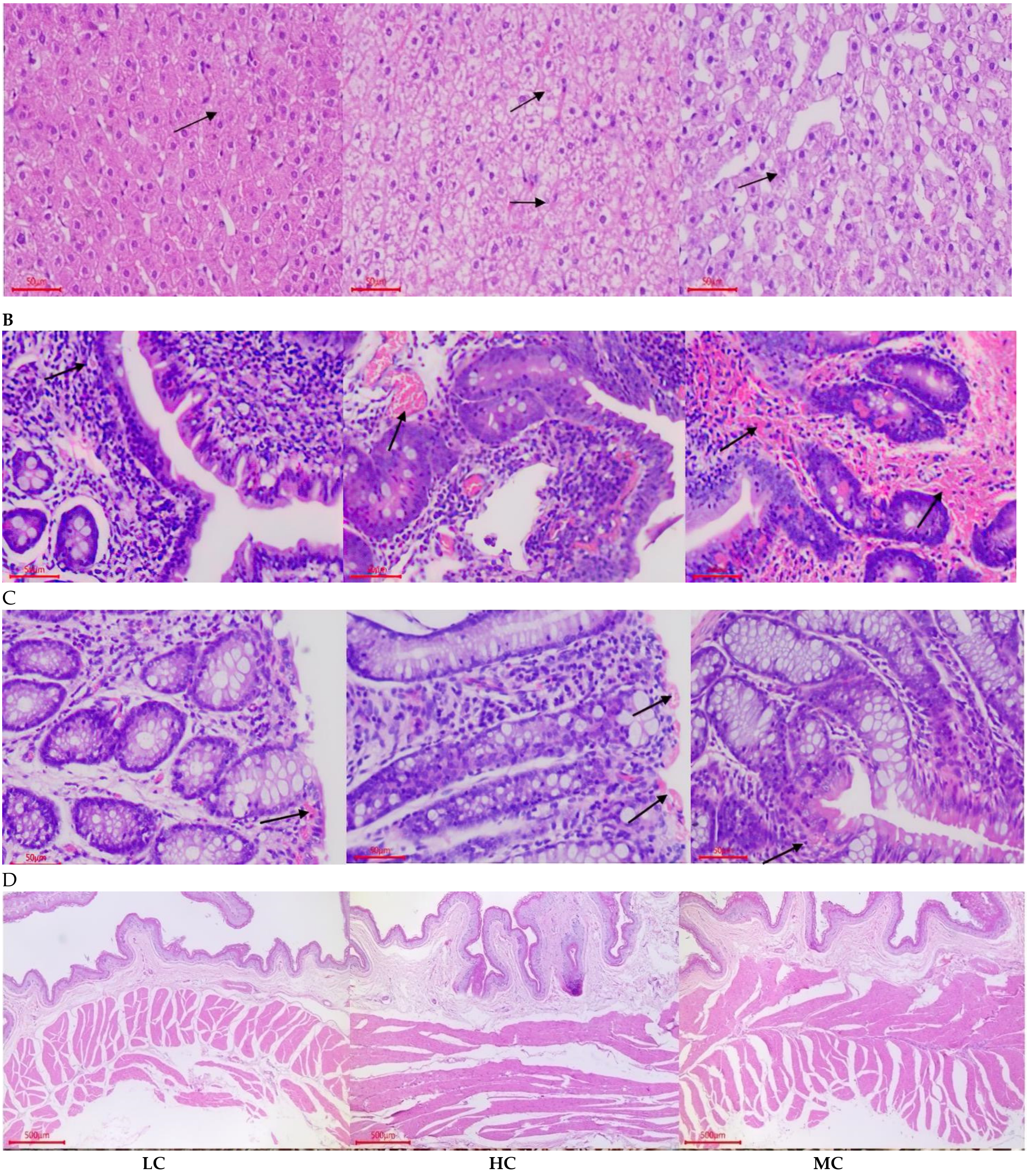

Figure S1. Different histopathological sections of goats (Bar=50μm，200x). (A) Pathological section of liver in goats of different groups; (B) Pathological section of jejunum in goats of different groups; (C) Pathological section of colon in goats of different groups; (D) Pathological section of rumen in goats of different groups. HC = high level of concentrate diet group; MC = medium level of concentrate diet group; LC= low level of concentrate diet group.

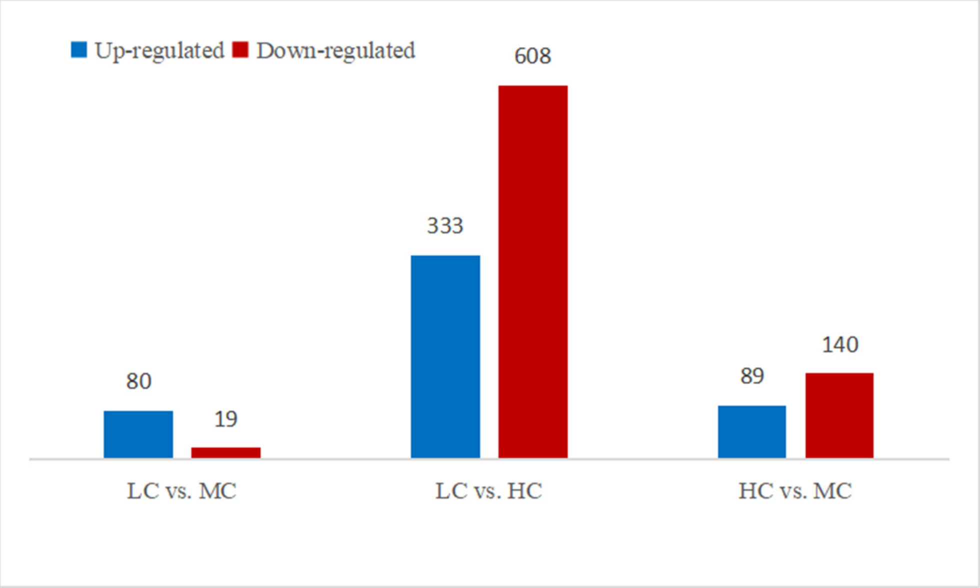

Figure S2. Numbers of differential expressed genes between two groups. HC = high level of concentrate diet group; MC = medium level of concentrate diet group; LC = low level of concentrate diet group.

2. *Tables*

Table S1. Primer pairs for real-time qPCR.

Gene name	Accession No.	Primer sequences (5′-3′)	Length
β-actin	NM_001314342.1	F: TCACGGAGCGTGGCTACAG<br>R: TTGATGTCACGGACGATTTCC	61bp
EIF4EBP1	NM_001285589.1	F:CCGGAGGTACCAGGATCATCT<br>R:CGTCTTGGTCACAGGTGAGTT	71bp
ACCS	XM_018059216.1	F: TGGCCAGGTTCCTGTCTTTCT<br>R: CCACCACATTCTCCGGTTTAAG	63bp
OXCT1	XM_013972785.2	F: CATTGCCAGTAAGCCGAGAGA<br>R: TGTGATTGCTTCCTCCAAGATAAAA	70bp
AACS	XM_005691340.3	F: CAGGTGTCTGGGCTCATGGT<br>R: ACGATTCCCCCGGTCTTG	58bp
APOA1	XM_018059749.1	F: GACCTTGGCTGTGCTCTTCCT<br>R: GGACGACTGTGGCTCATCTTG	73bp
APOC3	XM_005689483.3	F: CTACTCCTTCTTGCTGCCTTCCT<br>R: CCTCCTCGGCCTTGGTAGTT	64bp
APOA5	XM_018058993.1	F:CACGGAAAGGCTTCTGGGACT<br>R:CTCAAGTCTGTCTTTCAGGCTCG	110bp
HMGCR	XM_018053703.1	F: GCACGTCTACAGAAACTGCATATGA<br>R: TGGACTGAAAACGGATGTAAAGG	64bp

Table S2. Primer pairs for real-time qPCR.

Sample[1]	Clean Reads No.	Clean Data, bp	Clean Reads Rate, %
LC1	39174282	5876142300	93.7
LC2	40019798	6002969700	94.13
LC3	40645060	6096759000	93.69
LC4	43291734	6493760100	94.07
LC5	38813474	5822021100	93.51
LC6	42209404	6331410600	92.95
MC1	44133074	6619961100	93.5
MC2	38732456	5809868400	93.47
MC3	40571270	6085690500	93.93

[1]HC = high level of concentrate diet group; MC = medium level of concentrate diet group; LC = low level of concentrate diet group. Mapped Reads Rate = mapped reads/clean reads. Q30 value = bases of ≧ 30/all bases of sequencing.
